# A Novel Content and Usability Analysis of UK Professional Regulator Information About Raising a Concern by Members of the Public

**DOI:** 10.1111/hex.70027

**Published:** 2024-09-12

**Authors:** Gemma Ryan‐Blackwell, Louise M. Wallace, Francesca Ribenfors

**Affiliations:** ^1^ Faculty of Well‐being, Education & Language Studies The Open University Milton Keynes UK; ^2^ Department of Social Care & Social Work, Faculty of Health and Education Manchester Metropolitan University Manchester UK

**Keywords:** fitness to practise, navigability, patient complaints, professional regulation, qualitative content analysis, raising a concern, website usability

## Abstract

**Background:**

Health and social care regulators ensure that professionals have the correct qualifications and experience to practice in their profession. Globally, there are over 130 regulators of nursing alone and 13 health and social care statutory regulators in the United Kingdom. The public are the largest source of concerns to regulators about the registrants' fitness to practise (FtP).

**Aim:**

This study aimed to examine the amount, type and content of the information available from UK regulators and evaluate the usability of the process for members of the public considering raising a concern with a regulator about a registrant's fitness to practise (FtP) and the experience of those who had recently raised a concern.

**Methods:**

The websites of the UK's 13 statutory health and social care regulators were searched between November 2021 and February 2022 for information about the process of raising a concern. Webpages and public‐facing documentation were downloaded, and qualitative content analysis was conducted. The usability of regulator websites and the concerns referral form were assessed by 11 people using an adapted ‘system usability scale’. Seven interviews, a focus group (*n* = 5) and a survey (*n* = 62) of people who had raised a concern were used to explore their experiences to validate our findings and recommendations.

**Results:**

Themes were identified related to format and layout, the process and support to raise a concern, with wide variation found between regulators. Focus groups, interviews and surveys validated these findings.

**Discussion and Conclusion:**

Information and the ease of finding this information are fundamental in promoting public confidence and trust in regulator purpose and process. When raising a concern, it is important that information is honest, clear and accurate and available in a range of different formats so that it suits the diverse needs of members of the public. Improvements in these processes could support regulators to better achieve their primary purpose of protecting the public.

**Public Contribution:**

The public were consulted on our findings using two focus groups, seven interviews and 62 survey respondents.

Our project advisory group of people with lived experience of involvement in FtP discussed the findings and contributed to the recommendations.

**Trial Registration:**

N/A.

## 1 Background

Professional regulators are organisations that register members who have the correct qualifications and experience to practise their profession. Fitness to practise (FtP) refers to the health, character and competence to practise the profession [1]. Regulators contribute to patient safety by ensuring that professional standards are upheld, with the aim of protecting the public and maintaining public confidence in the profession [2]. Internationally, there are over 130 regulators of nurses [3] and over 118 regulators of doctors [4]. In the United Kingdom, there are 13 statutory health and social care regulators. The largest proportion of concerns raised with regulators is from members of the public [5, 6]. For example, in 2022, 75% of FtP concerns about doctors were from the public [6].

The provision and methods of delivering information are fundamental in promoting public trust in an organisation [5, 7, 8]. Therefore, if members of the public are to have confidence in regulators, their websites need to be easy to navigate with accurate and sufficient information for a member of the public who wishes to raise a concern [8, 9]. Aspects such as language, format, style, accuracy and transparency of information provided are shown to be essential in promoting confidence in institutions, in this case, health and social care regulators [8].

## 2 Literature Review

### 2.1 Concerns and Complaints

Regulators emphasise the need for the public and registrants to raise concerns about a professional's FtP to ensure the quality and safety of care [5, 7, 10].

In common use, a concern is ‘to relate to; be of importance or interest to; affect’ [11]. In relation to regulators, ‘escalating concerns is defined as taking a concern further by submitting evidence and going through the formal organisation processes’ [12]. Many regulators use the term concern and complaint interchangeably. However, in common use, a complaint is defined as ‘an objection to something that is unfair, unacceptable, or otherwise not up to normal standards’ [13] or in health services in the United Kingdom, ‘an expression of dissatisfaction that requires a response’ [14]. In order to distinguish those matters that would be resolved by a service provider, such as a registrant's employer, we use complaint to refer to matters reported to the provider of a service. We refer to concerns as matters raised with a regulator about professionals.

### 2.2 The Ease of Raising a Concern

Research into raising concerns has been conducted by some regulators [5, 7, 10]. Biggar et al. [15] conducted a large‐scale survey of both the public and registrants who raised concerns to an Australian healthcare regulator and found that 52% of participants found information easy to locate, but their results did not distinguish between the public and registrant responses. The General Medical Council (GMC) [7] commissioned workshops and interviews with members of the public and those who advocate on their behalf. They found that most raise concerns using regulators’ websites and noted that the content of information on the websites was important along with finding the right information with ease. They also found that there were challenges of raising a concern for those with protected characteristics, highlighting the need for independent support and advocacy services.

To undertake original and novel research on the websites of professional regulators, we utilised website usability methods.

### 2.3 Usability

Website usability means navigating the site to locate desired information, knowing what to do next and to do so with minimal effort [16].

For members of the public concerned about the behaviour of a registrant, the regulators’ websites contain important information on how to raise a concern, how it will be investigated and what happens if the concern reaches an FtP hearing. Seeking information and raising a concern about a registrant can take effort to recall and recount experiences that may have been physically or emotionally distressing and the person raising the concern may be feeling vulnerable at the time of raising the concern [5]. Therefore, being able to access the information they require and being able to raise the concern with ease are crucial [5]. It is known that users will abandon a task if they struggle to obtain the information required regardless of whether the website contains the information needed [9, 17]. Further, as technical ability in using websites is associated with higher levels of education [18], there is potential for differential exclusion of some groups, compounded by needing to access the websites in a language that may not be the person's first language [19].

Website usability and the content of information within a website are interdependent. Usability is supported by information provided in a manner that is fit for its purpose [20]. Usability is not solely about navigation, but website content also needs to be ‘usable’ for the purpose and audience for whom it is intended [9].

### 2.4 Aim

Our original research is the first to explore the provision of information by regulators on their websites for the public, including webpage content and the usability of the process up to the point of submitting a concern. It makes recommendations about what regulators can do to improve the experience of members of the public wanting to raise a concern.

### 2.5 Research Questions

i. What information do regulators provide for members of the public regarding raising a concern about a registrant?
ii. How easy is it for a member of the public to raise a concern to a regulator in the United Kingdom (UK) about a registrant?
iii. What are the experiences of members of the public when raising a concern?
iv. What can regulators do to improve processes for those who wish to raise a concern?

## 3 Methods

### 3.1 Design

A qualitative content analysis was applied to downloadable documents, webpages and videos on the 13 UK statutory professional regulator websites [21]. We carried out a usability test (quantitative and qualitative) of the process for raising a concern using regulator websites [22, 23, 24]. A focus group, interviews and a survey were used to explore people's expectations and experiences of raising a concern to validate our content analysis and usability findings and recommendations.

### 3.2 Data Collection

#### 3.2.1 Content Analysis

Data collection for the content analysis took place between November 2021 and February 2022 (this period was selected as this project was part of a larger programme of research about the public's role in fitness to practise). Webpages and downloadable documents were saved as portable download files (.pdf) and uploaded to the qualitative analysis software NVivo 12.0. Regulator websites were searched for information (including downloadable documents and webpages) relevant to the public about raising a concern. Information was excluded if the primary focus was on people who had already raised a concern (e.g., witness guidance; this information was analysed and has been reported on elsewhere) [25].

Resources were excluded if they were solely aimed at the registrant, expert witness or employers. Thirteen UK regulators were included (Table 1).

**Table 1:** The 13 statutory regulators.

Regulator	Acronym
General Chiropractic Council	GCC
General Dental Council	GDC
General Medical Council	GMC
General Optical Council	GOC
General Osteopathic Council	GOsC
General Pharmaceutical Council	GPhC
Health and Care Professionals Council	HCPC
Northern Ireland Social Care Council	NISCC
Nursing and Midwifery Council	NMC
Pharmaceutical Society of Northern Ireland	PSNI
Social Care Wales	SCW
Scottish Social Services Council	SSSC
Social Work England	SWE

#### 3.2.2 Survey

A cross‐sectional survey of public and colleague witnesses who raised a concern was conducted in 2022. Participants were identified by seven regulators on their databases as having raised a concern that was closed in the previous 6 months. Regulators sent a participant information sheet and link to an online consent form and survey, with contact details of the research team. Also, an invitation to contact the research team was shared via a flyer via the projects’ social media. Respondents’ concerns related to nine regulators. The survey included open‐ended questions about their experience of raising a concern (File S1).

#### 3.2.3 Focus Group and Interviews

A focus group and interviews were conducted to explore the content, format and usefulness of information offered to the public, including those who have previously raised a concern with a regulator. Recruitment was through patient participation groups of the GPhC and SWE, and a UK charity, Action against Medical Accidents (AvMA). Content was recorded and transcribed. As per our ethics approval, no personal or demographic data were collected, as these were anonymised. To be eligible to participate in the interviews, participants must have been adults over 18 years of age and a member of the public who had previously raised a concern with a regulator. Focus group participants were also required to be over 18 years of age and have lived experience of health or social care services.

Participants were asked to do a preparatory exercise that involved looking at several regulator documents and videos and answering questions about them that would prompt discussion in the interview or focus group. Interviewees from the AvMA explored their lived experience of the FtP process, including raising a concern and support needs [25, 26].

### 3.3 Analysis Methods

Qualitative content analysis as described in Altheide and Schneider [21] was applied to the websites, video transcripts and documents found. This method was used as it was specifically designed for qualitative analysis and derivation of meaning of different types of media/information. This is a 12‐step process that develops, tests and applies a coding framework. Stage five ‘tests’ the coding framework and at this point, it was identified that further data about the content of webpages were required. For example, what format were they in?

The survey comments, focus group and interview transcripts were tabulated using thematic analysis under high‐level themes derived from the content analysis [21].

#### 3.3.1 Assessment of Usability

We assessed the usability of the 13 regulator websites (Table 1); we applied a rating scale method. Since its inception, this SUS has been developed and become widely recognised as one of the most reliable and easy‐to‐use scales [22, 26].

The SUS contains 10 questions that are answered using a 5‐point Likert scale. For this project, an additional three questions were added (see File S2) to capture information about whether the participant achieved the goal of raising a concern. Text boxes were added below each rating to allow participants to explain their scores. These were important additions to ensure that we captured data reflecting the ISO 9241‐11 definition of usability, effectiveness, efficiency and satisfaction [22].

Participants were asked to approach the 13 regulators' *websites as a* ‘member of the public wanting to raise a concern about a professional’ through to the point of submitting their concern but not submitting the form. Participants included seven members of the research team (including a registered nurse, a registered social worker and a lay contributor who is also a patient advocate) and four registrant members of academic staff from the host university. These individuals were selected as a convenience sample and as time scales for the project did not allow for ethical approval to be obtained in time to recruit lay participants.

Participants completed the adapted SUS on the survey platform Qualtrics between May and October 2022. Knudson [22] recommends that at least 8–12 people complete the SUS for reliability.

#### 3.3.2 Analysis of Usability

Numerical responses to the original 10 SUS questions were scored using the SUS scoring system [23]. In four instances where a response was missing, the mid‐point of ‘not sure’ was selected [23]. The scoring system returns a single number (between 0 and 100) representing a composite measure of the overall usability of the website under investigation [23]. A score of 68 indicates above average usability and 80.3 indicates excellent usability [27]. A member of the research team collated, coded and grouped text responses within the survey.

In presenting the findings, Table 2 outlines the acronyms used for each data collection arm.

**Table 2:** Acronyms used to report on findings.

Data collection arm	Acronym
Focus group	FG
Survey participant	SP
Interview Participant	I
Usability tester	T

### 3.4 Ethical Considerations

The documents and webpages were in the public domain and the regulators were aware of the research. Ethical approval was granted by the Open University Human Research Ethics Committee (HREC/4058). Written consent was obtained from interview and focus group participants. The usability component did not require institutional ethics approval, as no personal or identifiable data were being collected.

## 4 Results

In total, 99 documents, webpages and video content were included in the analysis (File S3). The GMC (*n* = 19) and NMC (*n* = 14) had the highest number of resources and were the only regulators to have videos relating to the raising a concern process. SCW (*n* = 1) and GCC (*n* = 2) were regulators with the smallest number of resources (File S4).

File S5 provides numerical scores for usability. The websites with the two highest scores and, therefore, the most ‘usable’ were GCC (score = 72) and SCW (score = 72.5). None of the websites had an excellent score ( > 80.3). GOsC (score = 49) and NISCC (score = 49.44) had the lowest scores. The average range of scores across all regulators was 23.5, mean 58.6, s.d. 7.54.

### 4.1 Themes

Four main themes were identified through the content and usability analysis: (i) content, layout and format of information and webpages, (ii) the process of raising a concern, (iii) the ease of raising a concern and (iv) support available and alternative ways for raising a concern.

#### 4.1.1 Content, Layout and Format

##### 4.1.1.1 The Role of the Regulator: What Can and Cannot Be Investigated?

Twelve regulators provided some type of information about what can and cannot be investigated. Interviewees found this important for transparency and in setting expectations:

a bullet point list of that gave sort of guidance about what would be a complaint [concern], or you know that something that you could raise a complaint [concern] about.

(I1)

HCPC [28] also provided more detailed case examples about what can and cannot be investigated along with the outcome, which helped explain what was within their remit. This was endorsed by interviewees:

So having sort of short examples would actually be useful.

(I1)

The role of the regulator and an explanation of FtP were included by all regulators that interview participants welcomed:

Right, I would want to know what the regulator's actually, what their remit is, what their level of expertise is.

(I3)

A simply worded example is the GDC's easy‐read document *‘How to report a concern about a dentist or dental worker* [29].

Survey participants felt that the website content was useful but questioned whether it was trustworthy [8], as some felt that from their experience, this was not always the case:

With hindsight the regulator's website was NOT useful as they did not seem to follow their guidance as outlined in their own booklet.

(SP1)

The regulators website was useful, but what it said and what the HCPC did were different.

(SP2)

##### 4.1.1.2 Accessibility Webpages and Documents

Usability tests showed that regulators appeared to vary in the extent of adaptations taken to enhance accessibility. For example, some regulators had a prominent tab where language, font, colour, and so forth, could be changed to suit individual needs:

Excellent accessibility tool to cater for languages, disability, fonts, colours.

(T2)

The text of one website was considered very small, which could prove challenging for some people:

The writing was very small which could pose challenges to some people. It did say guidance documents were available in larger print, but this was written in small print.

(T1)

##### 4.1.1.3 Language

Usability testers appreciated when a tab including the word ‘concerns’ was clearly visible on the regulator's landing/home page. However, interviewees felt that as members of the public, they were not familiar with the term ‘fitness to practise’ and, therefore, they would find it difficult to locate information on raising a concern when it is included under that heading. Regulators sometimes switched between the use of concern and complaint—for consistency, it was suggested that it would be preferable to use the term ‘concern’ to distinguish this from complaints raised with an employer.

Finally, focus group participants felt that information should not include jargon and that plain English should be used rather than technical terms:

… but the documents have to be easy to read and in very … plain English. Legalistic terms, that's what would put people off straight away … so that would be a sort of disadvantage for many people.

(FG1)

##### 4.1.1.4 Audience

Some regulators provided different information and routes to different audiences when raising a concern. Some information was largely aimed at employers or managers even though situated in areas aimed at the public, which could suggest that members of the public are an afterthought or not as important. One interviewee suggested a solution:

Like if I go into a pharmaceutical website, they have little boxes up saying is this for a member of the public or are you a healthcare professional? So, you can work it that way.

(I3)

Having information early on clearly marked for members of the public was appreciated.

##### 4.1.1.5 Amount and Format of Information

Given the diversity of the general population, having the ‘right’ amount of information available for members of the public is a difficult balance for regulators to strike. Although some people in the usability tests, focus group and interviews appreciated detailed explanations and lots of information available, others found it overwhelming and confusing.

If they're crammed with too much information then they put you off, don't they?

(I3)

A simple flow chart of the concerns process and time frame was considered beneficial in addition to more thorough booklets to allow people to read what they felt they needed, which in turn can build trust [30]:

Yeah, the GMC website and what they do, and everything was very self‐explanatory. They'd got flowcharts about the process. It was very good, I thought the website was quite good, to be honest.

(I4)

Short pieces of information delivered via webpages were regarded as useful, and it is in line with UK Government [31] advice that webpages should be between 300 and 1250 words long to be most effective for the reader. The UK Government [32] also notes that although people read differently on webpages compared to when reading documents, they do not stipulate a minimum or maximum number of words (although Google [33] argues that a minimum of 300 is good practice from a search engine perspective).

Wienreich et al. [34] and the UK government [32] also suggest that users only read 20%–28% of a webpage and that webpages longer than 1250 words created ‘erratic’ reading patterns and became difficult to follow; for example, SCW [35] ‘How we deal with concerns’ was 1312 words long. Although it did provide some useful information, it could have been sectioned into smaller 300–500‐word pages so that the reader did not need to scroll down to search for specific information.

##### 4.1.1.6 Downloadable Documents

Some interviewees and focus group members referred to downloading information to read offline or print and make notes on and felt that this should be an option:

I think that you need a website which you can download documents which have all the information, because lots of people can't read online…but on a phone definitely PDFs a lot easier.

(FG2)

##### 4.1.1.7 Hyperlinks and Navigation

Where hyperlinks to other information were included, people preferred the links to open in new windows so that the information on the original page that they were on was not lost (although this may prove challenging on mobile devices):

You if you have to kind of repeatedly go back to the home page… {with} smartphones you go back to the home page and then you find out that you've gone out of the site. So yeah, having hyperlinks.

(I1)

Some regulators had broken hyperlinks, and the presence of too many hyperlinks was considered unhelpful and overwhelming by the usability testers.

There were lots of links which opened new pages in the browser and meant I lost my way a little. It would just be better to have the information in a logical order.

(T1)

Interviewees commented favourably when they felt that the website was simple to navigate, it was easy to find the section they wanted, information was laid out in a logical order and there was not too much ‘clutter’ or unnecessary links on pages.

Sites that have got arrows and boxes are usually quite useful aren't they and if they're crammed with too much information then they put off, don't they?

(I3)

I suppose like kind of things like government sites where you can have something at the at the top that has like you know you can skip sections if you if you don't if you want to read about something specifically.

(I1)

Usability testing found that it was helpful when regulators included a navigation panel on one side of the screen so that people could track their ‘journey’ through the website and see where they were in the information, what they had already read and what there was left to read:

Having a specific list of the journey steps on the left‐hand side which stayed visible when opening different pages of information really helped me and allowed me to easily to follow the pathway and open pages of information and not get lost in the site.

(T2)

Focus group members judged that sharing important information via short videos was helpful and enhanced accessibility:

But also, videos are a good idea as well because lots of people go to YouTube for instructions on practically anything.

(FG2)

However, regulators should consider their intended audience, as some videos suggested as useful for members of the public were presented from the registrant's perspective. Again, this may make a member of the public feel like an afterthought, which could impact on their willingness to trust the regulator with their concern [8]. Furthermore, all videos need to include a transcript to ensure that they are accessible to people who use screen readers and subtitles [36]. In some instances, these were lacking:

I think that it should have either a transcript or r subtitles or both cause in a way, if you're going to the effort of producing a video, you may as well make it as accessible to as many people as possible.

(I1)

#### 4.1.2 Process of Raising a Concern

All regulators provided information about how to raise a concern, but this varied in clarity and level of detail. Multiple routes to raise concerns, such as an online form, email or telephone, were viewed as good practice by members of the public in interviews, focus groups and usability testing:

I had emails and phone calls with one person which made me feel more comfortable and allowed me to ask questions.

(SP1)

It was helpful to speak to someone on the phone.

(SP4)

Several interview participants felt that regulators could make themselves more visible to the public, as it is only possible to raise a concern if someone knows who to raise it with:

Remember that not everybody has got access to a computer, posters, posters in libraries, posters in the High Street, really they're selling product aren't they in a sense.

(I3)

Eight regulators were explicit about what information and evidence would be required to raise a concern such as HCPC [37] *What information is needed to raise a concern?* This was useful to help people prepare and gather the information required.

All regulators provided information about what would happen after a concern is raised, in terms of getting a response, but few gave time scales for key decisions. This may help aid decisions to refer and to continue to engage [38].

This lack of specificity was particularly important for the interviewees who had raised a concern:

It was frustrating in the timescales they gave and also when we spoke to them about each step of the way it was very frustrating hearing that.

(I4)

#### 4.1.3 Ease of Raising a Concern and the Form

The usability analysis revealed that some regulators had hyperlinks to the reporting form clearly visible on the main landing/home page. However, in most cases, several pages of information had to be scrolled though, or multiple screening questions answered, before the form was accessible. For some people in our usability testing and interviews, this proved frustrating, particularly where people did not want to have to process lots of information; they just wanted to go straight to the form to report their concern.

There were numerous pages before you get to the form—lots of redirections to other channels but also a lot of repetition about their [regulators]'s role.

(T5)

Therefore, it is recommended that a link to the raising a concern form is clearly visible on either the home page or the main ‘concerns’ page.

It took me a while to find the link to actually start raising the concern which might be off putting to some—possibly better to have it at the top of the page although I appreciate that they want people to make sure it is a concern they can investigate before starting the process.

(T7)

Usability testing showed that people valued a short form that did not request too much information at this stage in the process. Prompts for what to include when explaining what happened were considered helpful:

There is too much information on the website and so discourages you from complaining. The form to complain is buried in the detail. I didn't find it friendly at all. It also conflates professional issues (support for the complained against) with the complainants’ issues.

(T10)

Although people valued the option to raise a concern through different methods, having to download a form to complete ‘offline’ was challenging and off‐putting:

A little less user friendly by having to download form to complete rather than having an online form.

(T7)

Through usability testing, we found that people thought that it was helpful to have the option to upload documents or other evidence to support their concern should they wish to do so, although this was not something offered by every regulator. Positive feedback was given by usability testers when regulators provided the option to save the form and return to it later, enabling the user to complete it over several sessions if they wished. However, not all regulators had this as an option.

Good that link to online complaint form was included on first page and gave you the option to save and come back to it within 30 days and you can upload correspondence.

(T6)

Limits on the word or character count on the reporting form were considered a hinderance as they may not allow enough space for someone to explain their concern in a way that they want to. Some regulators specified on the form that certain information was mandatory, for example, the registrant's name, registration number or the date of the incident.

Mandatory are not possible to overrule. Reason I did not complete it was it got stuck on save doctor details which were filled in as if I saw him locally and did not add the NHS trust's central address‐easily by public who don't know the NHS system of Trusts. So too much required information.

(T5)

People may not know registrant or employer information, and therefore, it can make it harder for them to raise their concerns. Similarly, some forms required an email address for the person raising the concern to proceed, which may prove a barrier to some people who do not regularly use email for correspondence:

The online form would not allow you to proceed unless you entered an email address which not everyone has.

(T1)

The presence of screening questions presented a mixed response in our usability tests. Although one person appreciated that they were useful from the regulator's perspective and could understand why they were there, others found them frustrating and ‘off‐putting.’ They made the process long‐winded, sometimes contained repetitive information about the regulator's role and there was a concern that they may act as a deterrent to people who had valid concerns to raise:

Raising a concern in a step‐by‐step process was easy to navigate. Layout (pictures and links in boxes) is better than the other websites I have used so far.

(T6)

The webform went into a loop at step 4 of 6 when raising my concern and did not respond after that so that I couldn't complete my form to raise a concern.

(T4)

This suggests that step‐by‐step screening/processes can be useful but only if structured carefully and simply so that it does not prolong the time taken to arrive at the form unnecessarily.

Avoiding repetition was viewed positively by survey participants, as they felt frustrated if asked to repeat their information several times:

It was a very time consuming and traumatic process. I had to explain myself several times.

(SP3)

There was a concern that binary gender options on the reporting form may not suit everyone. Indeed, they may deter people from reporting their concerns, particularly if their concerns contain gender‐related issues:

Only offers binary gender choice which may put some people off especially if complaining about gender related care.

(T7)

#### 4.1.4 Support Available/Alternative Ways for Raising a Concern

Eleven regulators signposted to other organisations to raise a complaint (rather than an FtP concern). An example is the HCPC [37] *How to make a complaint to the Health and Care Professionals Council*, as this site described each step, with short webpages providing information about what they can and cannot investigate, followed by signposts to different organisations complaints can be raised with and in what circumstances. There was some signposting to support independent of the regulator was also viewed positively by usability testers and interviewees:

Patients' Association, they've been really very, very good. There are also, for older people, there's I think Silver Thread which I've never used, but again they're very good. But Joe Public doesn't know. So, it's selling themselves to Joe Public.

(I3)

The GMC provided information about where else to go to make a complaint (rather than raise an FtP concern) or seek independent support, which participants interpreted as being informed about the ‘right’ place to go to achieve the desired outcome rather than reporting to the GMC incorrectly:

I think it's helpful to be directed as I suppose there's nothing kind of worse than feeling that you want to pursue something and then the left feeling that you don't know sort of how to go about doing that. So, I think that being directed to all the sources.

(I1)

All our participants commented favourably on the provision of clear information at the start of the process on the help available to raise a concern, such as a phone number to call:

By the information that they provided and the support that they gave, and any time I contacted them by telephone also I had the specific number for the investigator, and they always got back to me when I was making contact, if I needed them to contact me back, they were very good with that.

(I6)

The combination of website information and regulator telephone support was valued by participants:

*Both were equally informative and valuable when combined*.

(SP)

However, it was important to usability testers that the contact details were easy to find:

I had to download a leaflet which said I should call to complain but the number was not working.

(SP)

Four regulators allowed people to raise concerns anonymously and outlined the implications of doing this:

If you wish to remain anonymous you can still make a referral, however, please be advised that you will not receive any updates on the progress of the investigation. [39]

This is deemed helpful to members of the public in that it provides them with the choice about whether to remain anonymous and could reduce anxiety about raising a concern and what will happen afterwards. However, the process was not always as anonymous as it could be. For example, one regulator required an email, which, a usability tester noted that depending on the person's email address, may prevent it from being anonymous:

I could make an anonymous complaint by email but surely that is not anonymous completely?

(T1)

## 5 Discussion/Recommendations

Like the GMC report [7], our findings indicate that regulators would benefit from enhancing their public visibility so that people know where to go to when they want to raise a concern instead of having to search for information via different organisations. Hawkins [40] analysed the concept of public trust in organisations who regulate risk, outlining two concepts required for the public to trust an organisation: competence‐based trust (good reasons to trust that the organisation is able to do what it says it will) and motive‐based trust (the expectation that the organisation will do what it says). However, GMC [5, 7] and our findings suggest that regulator websites need to be more explicit about their role and purpose for the public to promote public trust. Our research makes clear that a focus on the public is essential in promoting public trust, with information that reassures people that they are ‘in the right place’ to raise their concern and that it will be taken seriously [8, 40]. To promote competence‐based trust [40], information should be transparent about what can and cannot be investigated by regulators with the use of case examples as well as bullet pointed lists for usability and accessibility purposes [41]. Information about what to expect after raising a concern, such as time frames and support during the process, was seen to be important in promoting motive‐based trust [40]. Flowcharts, videos and bullet‐pointed lists of complex information and processes were seen to be useful in addition to more detailed written information. The use of ‘symbolic’ content such as images, figures and other media assets has also been shown to make content more believable, facilitating people's trust in an organisation [8, 31]. This resonates with research on health information websites on the quality of content, accessibility and usability of health service website information for the public in Korea [42] and the United Kingdom [43].

The GMC [5, 7] established that the ease of raising a concern is a key driver for the public intending to raise a concern. It is known that intrinsic characteristics such as socioeconomic group, personality traits and perception and extrinsic factors such as society, organisations, policy and, in relation to our study, information and website usability may impact on people's willingness to trust [40, 44]. Addressing these drivers is important for regulators, as these influence the way people perceive and experience regulator websites and the information presented impacts on their confidence and trust in the regulator [31, 44, 45]. Our study found that it was not always an easy process, with the need for regulators to use non‐legalistic language, information in different formats for different needs (including those with protected characteristics) and the provision of a single point of contact to speak directly to someone at the regulator [7]. This in turn would improve public experience of and trust in the process from the outset [8].

### 5.1 Impact and Limitations

Although this study researched the 13 UK statutory regulators of health and social care professionals, there are 29 accredited registers (accredited registers work alongside employers, commissioners, local authorities, patient and consumer protection agencies as part of a quality assurance framework. They aim to promote confidence in practitioners who are not regulated by one of the 13 UK regulators, protect from risk and improve standards. Accredited registers are assessed and approved by the Professional Standards Authority [46]) in the United Kindom where these findings are applicable.

Our study is original and internationally relevant; it is the first study to have considered how regulators can promote public trust as part of the process of raising a concern. Internationally, there are hundreds of regulators globally whose role is public protection (not solely in health and social care) for whom these findings will be transferrable, providing data about how regulatory trust can be promoted by appropriately designed information about how concerns can be raised.

We found that most research focuses on medicine or dentistry, and is not commissioned by a regulator and/or does not examine the public experience of raising an FtP concern. A strength of our novel study is that it is the first multiregulator study independent of regulators, exploring the public experience of raising a concern to a regulator. No other studies have used robust and reproducible methods for this purpose, triangulated this with data from the public and those who have made FtP referrals and assessed the usability of concern submission processes for members of the public.

A limitation is the use of a sample of professionals to conduct the usability analysis. A future study could include a more diverse sample, particularly those who might need support to engage with the processes.

## 6 Conclusion

Regulators seek to protect the safety of the public and, in doing this, are required to promote public trust and confidence in FtP processes. The provision of information and the ease of use of this information are known to be fundamental components in promoting public confidence and trust in an organisation. Our study found that relevant, accurate and clear information available that is delivered in different formats, such as using videos and figures and images (such as flowcharts) to summarise complex information, is important when raising a concern. Furthermore, how webpages and documents are presented are also important for usability and in improving the ease of raising a concern. The different types of information available and webpages should ensure that the known barriers to effective communication such as language, disability and digital literacy are addressed. It is also important that regulators promote their prime purpose of public protection, their responsibility and mission to the public and information for the public should focus on the publics' need to instil confidence and trust that they will take concerns seriously and that their processes are robust and for public.

## Author Contributions

**Gemma Ryan‐Blackwell:** conceptualisation, investigation, funding acquisition, writing–original draft, methodology, validation, writing–review and editing, formal analysis, project administration, data curation. **Louise M. Wallace:** conceptualisation, investigation, funding acquisition, writing–original draft, validation, supervision, writing–review and editing. **Francesca Ribenfors:** investigation, writing–original draft, writing–review and editing, project administration, formal analysis, data curation, methodology.

## Ethics Statement

The Open University Human Research Ethics Committee HREC/4058.

## Consent

Participants provided valid informed consent.

## Conflicts of Interest

Louise Wallace is a Lay Panel member for the General Dental Council and Lay Adjudicator for Social Work England.

## Supporting information

Supporting information.

Supporting information.

Supporting information.

Supporting information.

Supporting information.

## Data Availability

Data from this research are available by contacting the corresponding author.
