# Supplementary material for: A Novel Content and Usability Analysis of UK Professional Regulator Information About Raising a Concern by Members of the Public
Source: Health Expect. 2024 Sep 12;27(5):e70027. doi: 10.1111/hex.70027 (PMC11391942; doi:10.1111/hex.70027)
Supplement: Supplementary file 1 — Supporting information. [file HEX-27-e70027-s002.docx]

Survey questions about raising a concern

What information sources did you have about the purpose and what you might need to do in the fitness to practise process?  (Tick all that apply)

- Information from regulator’s website or from the regulator’s staff

- Citizen’s Advice,

- HealthWatch

- AvMA(Association for Victims of Medical Accidents)

- Victim Support,

- Patient Advice and Liaison Service (PALS)

- a lawyer

- Other- please explain

What sources of information were most useful and why? (OPEN question)

What sources of personal support were offered by the regulator? (Tick all that apply)

- None

- Witness support officer/service of the regulator

- One person as point of contact in the regulator

- An independent Victim/Witness Support counsellor

- Other-please explain

Did you make use of any sources of support from the regulator or others after the case was closed?  (Tick all that apply)

- No

- Witness support officer/service of the regulator

- One person as point of contact in the regulator

- An independent Victim/Witness Support counsellor

- Other-please explain (TEXT BOX)

How helpful was this source of support? (tick one)

- Very helpful

- Helpful

- Not helpful

- Very unhelpful

Comments:

What could be improved by the regulator in relation to the witness’s experience of Fitness to Practise proceedings?

(OPEN QUESTION)

Would you/others like you in future benefit from publicly available information and advice independent of the regulator?  Yes/No/Don’t know

(OPEN QUESTION)
