# Supplementary material for: A Novel Content and Usability Analysis of UK Professional Regulator Information About Raising a Concern by Members of the Public
Source: Health Expect. 2024 Sep 12;27(5):e70027. doi: 10.1111/hex.70027 (PMC11391942; doi:10.1111/hex.70027)
Supplement: Supplementary file 2 — Supporting information. [file HEX-27-e70027-s001.docx]

## Website usability questionnaire from Chiew & Salim (2003)^1^

1. Chiew TK, Salim SS. *WEBUSE: WEBSITE USABILITY EVALUATION TOOL*. Vol 16.; 2003.

### Evaluating content, organisation & readability

§ This website contains most of my interest material and topics and they are up-to-date.

§ I can easily find what I want at this website.

§ The content of this website is well organised.

§ Reading content at this website is easy.

§ I am comfortable and familiar with the language used.

§ I need not scroll left and right when reading at this website.

### Evaluating navigation and links

§ I can easily know where I am at this website.

§ This website provides useful cues and links for me to get the desired information.

§ It is easy to move around at this website by using the links or back button of the browser.

§ The links at this website are well maintained and updated.

§ The website does not open too many new browser windows when I am moving around.

§ Placement of links or menu is standard throughout the website and I can easily recognise them.

### Evaluation of user interface

§ This website’s interface design is attractive.

§ I am comfortable with the colours used at this website.

§ This website contains no feature that irritates me such as scrolling or blinking text and looping

animations.

§ This website has a consistent feel and look.

§ This website does not contain too many Web advertisements.

§ The design of the website makes sense and it is easy to learn how to use it.

### Performance and effectiveness

§ I need not wait too long to download a file or open a page.

§ I can easily distinguish between visited and not-visited links.

§ I can access this website most of the time.

§ This website responds to my actions as expected.

§ It is efficient to use this website.

§ This website always provides clear and useful messages when I don’t know how to proceed.

## Design

1. Did you like the design of the website?
2. Did you like the colors of the website?
3. Were you able to differentiate links easily?
4. Did you find the images on the website useful?
5. Did you find the images on the website relevant?
6. How was the experience with our website on your smartphone?
7. Was the size of web controls appropriate enough?
8. Could you get all the necessary features on our mobile website?
9. Do you think you had to click too much to get what you were looking for?
10. Did the website render well in your browser?
11. On a scale of 0-10, how would you rate the usability of our website?
12. What do you like about our existing website?
13. What would you like to change on our existing website?
14. What would you like to see on our new website?

## Navigation

1. Were you able to find links easily?
2. Were you able to navigate to other pages easily?
3. Did the links take you to the relevant pages?

## Content

1. Did you find this page meaningful?
2. Did you find the content on the website relevant?
3. Did this page help you?
4. Could you search the content you were looking for?
5. On a scale of 1-10 how do you rate the clarity of the content?
6. On a scale of 1-10 how do you rate the conciseness of the content?

## Performance

1. Did it take too long to load the website?
2. Did it take too long to fetch your details on our website?
3. Did the saving of your data take too long?
4. On a scale of 1-10, how would you rate the performance of our website?

## General

1. What do you like most about our website?
2. How often do you visit our website?
3. What would you like to change in the website?
4. On a scale of 1-10, how likely are you to recommend our website to your friends or family?
5. Did you face any challenge while using our website?
6. Do you have any suggestions or comments?
