# Supplementary material for: A Novel Content and Usability Analysis of UK Professional Regulator Information About Raising a Concern by Members of the Public
Source: Health Expect. 2024 Sep 12;27(5):e70027. doi: 10.1111/hex.70027 (PMC11391942; doi:10.1111/hex.70027)
Supplement: Supplementary file 3 — Supporting information. [file HEX-27-e70027-s004.docx]

A summary of included resources by type and regulator

| **Regulator** | **Number of downloadable leaflets (e.g. pdfs/word docs)** | **Number of documents created from webpages** | **No. of videos** | **Number of easy read documents** | **Total resources** |
| --- | --- | --- | --- | --- | --- |
| **GCC** | 0 | 2 | 0 | 0 | ***2*** |
| **GDC** | 2 | 10 | 0 | 1 | ***13*** |
| **GMC** | 0 | 17 | 1 | 1 | ***19*** |
| **GOC** | 1 | 4 | 0 | 0 | ***5*** |
| **GOsC** | 1 | 3 | 0 | 0 | ***4*** |
| **GPhC** | 3 | 8 | 0 | 0 | ***11*** |
| **HCPC** | 1 | 9 | 0 | 1 | ***11*** |
| **NISCC** | 1 | 2 | 0 | 0 | ***3*** |
| **NMC** | 3 | 8 | 1 | 2 | ***14*** |
| **PSNI** | 1 | 2 | 0 | 0 | ***3*** |
| **SCW** | 0 | 1 | 0 | 0 | ***1*** |
| **SSSC** | 1 | 3 | 0 | 0 | ***4*** |
| **SWE** | 0 | 9 | 0 | 0 | ***9*** |
| ***Total*** | ***14*** | ***78*** | ***2*** | ***5*** | ***99*** |
