# Supplementary material for: A Novel Content and Usability Analysis of UK Professional Regulator Information About Raising a Concern by Members of the Public
Source: Health Expect. 2024 Sep 12;27(5):e70027. doi: 10.1111/hex.70027 (PMC11391942; doi:10.1111/hex.70027)
Supplement: Supplementary file 4 — Supporting information. [file HEX-27-e70027-s005.docx]

A summary of content found/topics covered across all formats by regulator

| **Stage** | **Topic** | **GCC** | **GDC** | **GMC** | **GOC** | **GosC** | **GPhC** | **HCPC** | **NISCC** | **NMC** | **PSNI** | **SCW** | **SSSC** | **SWE** | ***Total*** |
| --- | --- | --- | --- | --- | --- | --- | --- | --- | --- | --- | --- | --- | --- | --- | --- |
| Before making concern | What is FtP/role of regulators? | ✓ | ✓ | ✓ | ✓ | ✓ | ✓ | ✓ | ✓ | ✓ | ✓ | ✓ | ✓ | ✓ | ***13*** |
|  | What can and cannot be investigated? | ✓ | ✓ | ✓ | ✓ | ✓ | ✓ | ✓ | ✓ | ✓ |  | ✓ | ✓ | ✓ | ***12*** |
|  | Regulator support (contact details for someone to speak to) | ✓ | ✓ | ✓ | ✓ | ✓ | ✓ | ✓ | ✓ | ✓ | ✓ | ✓ | ✓ | ✓ | ***13*** |
|  | Independent support/raising a concern elsewhere |  | ✓ | ✓ | ✓ |  | ✓ | ✓ | ✓ | ✓ | ✓ | ✓ | ✓ | ✓ | ***11*** |
| Raising a concern | How to raise a concern | ✓ | ✓ | ✓ | ✓ | ✓ | ✓ | ✓ | ✓ | ✓ | ✓ | ✓ | ✓ | ✓ | ***13*** |
|  | Regulator support | ✓ | ✓ | ✓ | ✓ | ✓ | ✓ | ✓ | ✓ | ✓ | ✓ | ✓ | ✓ | ✓ | ***13*** |
|  | Independent support |  | ✓ | ✓ | ✓ |  | ✓ | ✓ | ✓ | ✓ |  |  |  | ✓ | ***8*** |
|  | What happens after raising a concern? e.g. a summary of the process | ✓ | ✓ | ✓ | ✓ | ✓ | ✓ | ✓ | ✓ | ✓ | ✓ | ✓ | ✓ | ✓ | ***13*** |
| ***Total*** | | ***6*** | ***7*** | ***8*** | ***8*** | ***6*** | ***8*** | ***8*** | ***8*** | ***8*** | ***6*** | ***7*** | ***7*** | ***8*** |  |
