# Supplementary material for: A Novel Content and Usability Analysis of UK Professional Regulator Information About Raising a Concern by Members of the Public
Source: Health Expect. 2024 Sep 12;27(5):e70027. doi: 10.1111/hex.70027 (PMC11391942; doi:10.1111/hex.70027)
Supplement: Supplementary file 5 — Supporting information. [file HEX-27-e70027-s003.docx]

*Numerical results from SUS*

|  | 1 | 2 | 3 | 4 | 5 | 6 | 7 | 8 | 9 | 10 | 11 | ***Mean*** |
| --- | --- | --- | --- | --- | --- | --- | --- | --- | --- | --- | --- | --- |
| **GCC** | 87.5 | N/A | 25 | 92.5 | 72.5 | 77.5 | 80 | 67.5 | 57.5 | 75 | 85 | ***72*** |
| **GDC** | 70 | 52.5 | 30 | 45 | 47.5 | 30 | 75 | 62.5 | 65 | 32.5 | 72.5 | ***52.95*** |
| **GMC** | 45 | 40 | 22.5 | 55 | 67.5 | 35 | 60 | 67.5 | 35 | 75 | 72.5 | ***52.27*** |
| **GOC** | 47.5 | 35 | 40 | 60 | 72.5 | 35 | 75 | 67.5 | 72.5 | 75 | 75 | ***59.54*** |
| **GOsC** | 50 | 75 | 50 | 32.5 | 40* | 45 | 52.5 | 42.5 | 72.5 | N/A | 30 | ***49*** |
| **GPhC** | 80 | 37.5 | 35 | 72.5 | 65 | 77.5 | 80 | 70 | 75 | N/A | 50 | ***64.25*** |
| **HCPC** | 47.5 | 77.5 | 45 | 30 | 62.5 | 87.5 | 80 | 70 | 25 | N/A | 90 | ***61.5*** |
| **NISCC** | 47.5 | 12.5 | 45 | 57.5 | 32.5* | 32.5 | 77.5 | 67.5 | N/A | N/A | 72.5 | ***49.44*** |
| **NMC** | 37.5 | 70 | 20 | 90 | 62.5 | 50 | 80 | 67.5* | N/A | N/A | 75 | ***61.38*** |
| **PSNI** | 32.5 | 87.5 | 25 | 42.5 | 42.5 | 57.5 | 70 | 60 | N/A | N/A | 57.5 | ***52.77*** |
| **SCW** | N/A | 92.5 | 35 | 87.5 | 67.5 | 82.5 | 62.5 | 70 | N/A | N/A | 82.5 | ***72.5*** |
| **SSSC** | 35 | 30* | 35 | 72.5 | 60 | 55 | 70 | 65 | N/A | N/A | 55 | ***53.05*** |
| **SWE** | 75 | 22.5 | 35 | 95 | N/A | 47.5 | 67.5 | 67.5 | N/A | N/A | 75 | ***60.62*** |

**Indicates where a response was missing and the mid-point of ‘not sure’ was substituted*

*N/A indicates where a participant did not fully complete the SUS for that regulator*
